# In Vivo Evidence of Myofascial Force Transmission Along the Posterior Spiral Chain: Functional Connectivity Linking the Contralateral Latissimus Dorsi, Thoracolumbar Fascia, and Gluteal Region

**DOI:** 10.7759/cureus.100760

**Published:** 2026-01-04

**Authors:** Saverio Colonna, Greta Maietti, Federico Cuoghi

**Affiliations:** 1 Rehabilitation Medicine, Spine Center, Bologna, ITA; 2 Research and Development, Osteopathic Spine Center Education, Bologna, ITA

**Keywords:** biomechanics, functional connectivity, in vivo study, muscle energy technique, myofascial chains, myofascial force transmission, posterior spiral chain, range of motion, thoracolumbar fascia, trunk rotation

## Abstract

Myofascial force transmission has been proposed as a mechanism by which mechanical stimuli applied to one body region can influence the behavior of distant segments through fascial continuity. The posterior spiral chain (PSC), linking the latissimus dorsi, thoracolumbar fascia, and contralateral gluteal region, has been described anatomically, but its functional relevance in vivo remains incompletely understood.

A single-group pre-post experimental study was conducted on 73 healthy participants. Trunk rotation in the transverse plane was assessed using inertial digital goniometry under four conditions: two baseline assessments, isometric activation of the PSC via right hip abduction-external rotation, and post-stretching of the caudal segment of the PSC (right or left side, alternating allocation procedure). Left and right trunk rotation, total rotational range of motion (ROM), and rotational balance were analyzed.

Baseline trunk rotation showed no lateral asymmetry and no sex-related differences. Isometric activation of the PSC induced a marked redistribution of trunk rotation, characterized by a significant increase in left rotation and a concomitant reduction in right rotation, with only a small reduction in total ROM. Stretching produced a partial rebalancing of the activation-induced asymmetry. While total ROM did not differ between stretching sides, rotational balance shifted toward the treated side, indicating a direction-specific effect.

Modulation of myofascial tension along the posterior spiral chain alters trunk rotational patterns in vivo without increasing overall rotational amplitude. These findings support the functional relevance of the PSC and suggest that myofascial interventions may primarily act by redistributing available motion rather than by increasing total range of motion, with potential implications for the assessment and treatment of rotational dysfunctions.

## Introduction

The fascial system is currently regarded as a continuous anatomical network capable of connecting even distant body regions and contributing to the transmission and modulation of muscular forces. Experimental studies have shown that a substantial proportion of the force generated by muscle contraction is not transmitted exclusively through the myotendinous junction but also via intra-, inter-, and extramuscular myofascial pathways, potentially affecting structures that are not directly adjacent to the activated muscle [1-4]. 

Fascial tissue exhibits sufficient stiffness to transmit approximately 15% to 37% of muscular force to adjacent structures rather than conveying it entirely to the tendinous insertion [5]. The magnitude of this force transmission depends on the length and relative spatial arrangement of the involved tissues [6,7]. Moreover, these forces may be distributed over a distance, thereby influencing the range of motion of segments remote from the initial site of muscular contraction [8]. This long-distance phenomenon has been defined by Wilke et al. [9] as the “remote effect,” referring to biomechanical or functional changes observed in body regions other than the one directly subjected to treatment. Accordingly, a related hypothesis is that myofascial connectivity contributes to the effects of “remote stretching” through force transmission along myofascial chains. Given the rich innervation of these tissues, the term "neuro-myofascial systems" may be more appropriate.

A systematic review by Wilke et al. [9] provided strong evidence, primarily based on anatomical dissection studies, for the existence of at least three myofascial chains proposed by Myers [10]: the superficial back line, the back functional line, and the front functional line. In contrast, the evidence supporting the spiral line and the lateral line was rated as moderate to strong but less consistent.

Studies investigating remote effects have predominantly focused on myofascial systems with a mainly sagittal orientation [9,11-17], particularly the superficial back line. When examining the myofascial connectivity models proposed in the literature, all authors [10,18-20] agree on the existence of a posterior sagittal antigravitational system connecting the cranial and podalic regions bilaterally. However, not all models describe a system linking the right cranial region with the contralateral (left) podalic region. Among the systems involved in rotational body mechanics, Busquet [18] refers to these connections as crossed, Myers [10] defines them as functional, while Stecco [19] and Colonna [21] describe them as spiral, emphasizing their three-dimensional spatial organization.

In contrast to the interpretations proposed by Stecco [19] and Colonna [21], the spiral line described by Myers [10] appears to be more difficult to interpret biomechanically, as it is conceptualized as a concatenation of an anterior spiral component that continues into a posterior sagittal component.

Within the framework of posterior spiral chains, the thoracolumbar fascia (TLF) represents a crucial biomechanical hub. Its anatomical connections with the latissimus dorsi (LD) and the gluteus maximus (GMax) create a force-transmission pathway that crosses the lumbosacral region and functionally links the shoulder girdle to the contralateral lower limb [22]. Cadaveric anatomical studies have shown that tension applied to either the LD or the GMax produces measurable displacements within the TLF, including on the contralateral side, supporting the existence of a true mechanical continuity along this crossed posterior pathway [23,24].

Within this context, the concept of the posterior spiral chain (PSC) provides a three-dimensional functional interpretation of this system: a myofascial pathway connecting the upper limb through the latissimus dorsi, the thoracolumbar fascia, and the contralateral lower limb via the gluteal region [25]. This model integrates features of the crossed chains described by Busquet [18] and the “functional lines” reported in the literature [10].

The PSC is particularly involved in trunk rotation tasks and in movements requiring crossed pelvic stabilization, in which one myofascial spiral acts as the functional agonist of rotation while the contralateral spiral must effectively lengthen to serve as a functional antagonist. For example, during left trunk rotation, active engagement of the right anterior spiral and the left posterior spiral must be accompanied by adequate relaxation of the opposing chains, namely, the left anterior spiral and the right posterior spiral, to avoid mechanical restriction of movement.

In vivo evidence has begun to support this model. Carvalhais et al. [23] demonstrated that increasing tension in the latissimus dorsi can modify the passive mechanical properties of the contralateral hip, providing early experimental support for myofascial force transmission along what has been described as the “back functional line” [1,10,26], the “posterior oblique sling” [22], or the posterior spiral chain [19,21,27]. More recent studies in runners have shown that latissimus dorsi contraction is associated with changes in lumbar stiffness and passive characteristics of the contralateral hip, further reinforcing the hypothesis of a functional LD-TLF-GMax coupling [23,25].

Despite the growing body of anatomical and experimental evidence on myofascial force transmission, the in vivo functional relevance of this crossed posterior chain remains insufficiently clarified. In particular, it is still unclear whether, and to what extent, modulation of tension at the caudal end of the PSC, for instance through isometric contraction or stretching of the hip abductors and external rotators, can alter trunk rotational range of motion in the transverse plane without changing the overall movement amplitude, but rather by redistributing its directional expression [1].

The aim of the present study was to test this hypothesis by investigating whether an increase in active tension (via isometric contraction) and/or a reduction in passive tension (via muscle energy stretching techniques) at the caudal segment of the PSC induces measurable changes in trunk rotational range of motion in the transverse plane. Demonstrating such changes in the absence of modifications in total rotational amplitude would provide further in vivo evidence of myofascial force transmission along the PSC and support its functional role in the regulation of trunk rotation and postural spirals [23,25].

## Materials and methods

Study design

A single-group pre-post experimental study was conducted to investigate the effects of myofascial tension modulation at the caudal segment of the Posterior Spiral Chain (PSC) on trunk rotational range of motion in the transverse plane. Each participant completed four testing conditions in the following order: Baseline 1; Baseline 2; Isometric activation of the right PSC; Post-stretching of the PSC (right or left side, randomly assigned). All measurements were obtained using inertial digital goniometry.

Participants

Seventy-five healthy participants (48 males, 27 females) were initially recruited-all students from the OSCE school (Osteopathic Spine Center Education); two participants were excluded a posteriori due to acquisition errors related to device calibration. Therefore, data from 73 participants were included in the final analysis.

Participants were aged between 18 and 40 years and had no history of spinal disorders that could limit trunk rotation. Exclusion criteria included the presence of radicular pain or neurological signs, significant spondylolisthesis, spinal stenosis, structural scoliosis, or a history of spinal surgery.

Instrumentation

Trunk rotation was assessed using the Free4Act system (F4A LetSense Srl, Bologna, Italy), a wireless digital goniometer based on inertial sensors. This system has been validated in the literature for the analysis of trunk and pelvic motion [28,29].

Participant positioning and movement standardization

Across all experimental conditions, participants were seated on the edge of an examination table with hips and knees flexed at 90°, feet fully supported on the floor, and a bar positioned across the shoulders and held with the forearms. Pelvic motion was manually controlled by the examiner using a standardized hand placement to minimize compensatory movements. All assessments were performed by the same trained examiner to ensure procedural consistency across participants. Examiner positioning and pelvic stabilization are illustrated in Supplementary Appendix 1.

During the activation condition, isometric activation was consistently performed involving the right lower limb in order to standardize the direction of myofascial tension along the posterior spiral chain across participants.

Stretching was performed using a modified muscle energy technique (MET) targeting the caudal segment of the PSC, as described above. The side of stretching was assigned using a systematic alternating allocation procedure: the first participant underwent stretching on the left side, and subsequent participants were alternately assigned to right and left stretching in sequence. This approach was adopted to ensure a balanced distribution of stretching laterality across the sample.

A detailed description of participant positioning, trunk rotation execution, activation protocol, and stretching procedures is provided in Supplementary Appendix 1.

Statistical analysis

Normality of the data was assessed using the Shapiro-Wilk test. Since all primary outcomes were normally distributed, parametric statistics were applied. Paired t-tests were used to compare within-subject conditions (baseline vs. activation; left vs. right rotation), while independent-samples t-tests (with Welch’s correction when appropriate) were used for between-group comparisons (sex, limb dominance, stretching side).

No formal correction for multiple comparisons was applied. Given the exploratory design of the study and the presence of predefined, hypothesis-driven comparisons, results were interpreted using p-values in conjunction with effect sizes (Hedges’ g) and 95% confidence intervals.

Statistical significance was set at p < 0.05 (two-tailed), and effect sizes with 95% confidence intervals were used to support interpretation.

Standardized effect sizes were estimated using Hedges’ g rather than Cohen’s d, as Hedges’ g provides a bias correction for small sample sizes and unequal group distributions [30,31].

In the absence of robust preliminary data allowing reliable a priori sample size estimation, the study was designed using an adaptive approach [32]. An exploratory analysis of the first 30 participants revealed medium-to-large effects (Hedges’ g ≈ 0.7-0.8) for the primary outcomes related to trunk rotation changes during muscle activation. Based on these estimates, a paired t-test with α = 0.05 and 90% power would have required approximately 30 participants. To improve the robustness of subgroup analyses, recruitment was therefore continued up to a total sample of 75 participants.

## Results

Data from 73 participants (48 males and 27 females) were analyzed. The mean age was 24.03 ± 3.95 years, with a mean body weight of 69.06 ± 12.91 kg and a mean height of 171.87 ± 7.31 cm.

Shapiro-Wilk testing confirmed normal distribution of baseline trunk rotation values, supporting the use of parametric statistical analyses.

Baseline trunk rotation showed no lateral asymmetry (Table 1), confirming the absence of a baseline rotational bias.

**Table 1:** Baseline trunk rotation values were calculated as the mean of two repeated baseline assessments (Baseline I and Baseline II). Values are reported as mean ± standard deviation. A paired t-test was used to compare left and right rotation. Effect size is reported as Hedges’ g, and confidence intervals represent the 95% CI of the mean difference (left minus right).

Parameter	Left Rotation (°)	Right Rotation (°)	Mean Difference (Left / Right)	t-value	p-value	Hedges’ g	95% CI (Difference)
Baseline	57.60 ± 11.02	57.80 ± 12.05	–0.20°	–0.32	0.749	–0.04	–2.27 to +1.64

No sex-related differences were observed in baseline trunk rotation (Table 2). Accordingly, the sample was considered homogeneous with respect to sex-related baseline trunk rotational mobility.

**Table 2:** Baseline trunk rotation values stratified by sex. Means and standard deviations are presented for left and right rotation. Independent-samples t-tests were used to compare males and females. Effect sizes are reported as Hedges’ g, and 95% confidence intervals refer to the difference in means (male minus female). No statistically significant differences were observed between sexes.

Rotation	Female (n = 26)	Male (n = 47)	t-value	p-value	Hedges’ g	95% CI of Difference (Male – Female)
Left Rotation (°)	57.03 ± 11.31	57.91 ± 10.97	0.321	0.750	0.08	–4.62 to 6.37
Right Rotation (°)	56.57 ± 12.56	58.48 ± 11.85	0.635	0.529	0.16	–4.14 to 7.96

Baseline trunk rotation did not differ significantly between right- and left-dominant individuals (Table 3). Left-dominant participants tended to show higher rotation values in both directions; however, these differences did not reach statistical significance. The small number of left-dominant participants (n = 6) limited the statistical power of this analysis.

**Table 3:** Baseline trunk rotation in right- and left-dominant individuals. Values are reported as mean ± standard deviation. Group comparisons were performed using Welch’s t-test. Effect size is reported as Hedges’ g. Confidence intervals refer to the difference in means (right-dominant minus left-dominant). No statistically significant differences were observed.

Rotation Parameter	Right-Dominant (n = 67)	Left-Dominant (n = 6)	t-value	p-value	Hedges’ g	95% CI (Right – Left)
Left Rotation (°)	56.88 ± 10.41	65.54 ± 15.41	–1.35	0.231	–0.79	–24.78 to +7.47
Right Rotation (°)	56.90 ± 11.09	67.84 ± 18.37	–1.44	0.207	–0.92	–30.17 to +8.29

**Figure 1:**
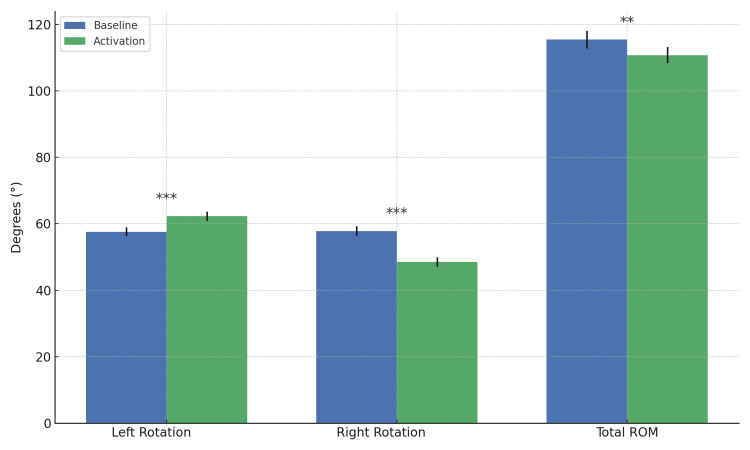
Baseline vs. activation trunk rotation (left, right, total ROM). Bar chart showing left rotation, right rotation, and total trunk rotation range of motion (ROM) at baseline and during activation. Bars represent mean values with standard error of the mean (SEM). Asterisks indicate statistical significance of paired comparisons: ***p < 0.001, **p < 0.01, *p < 0.05, and ns = not significant.

Muscle activation induced a marked redistribution of trunk rotational mobility (Tables 4-6). Compared with baseline, activation was associated with a significant increase in left rotation and a concomitant reduction in right rotation, resulting in a pronounced leftward shift in rotational capacity (Figure 1). Total trunk rotational ROM showed only a small change, indicating that activation primarily altered the directional distribution of rotation rather than producing a substantial net gain or loss of overall mobility.

**Table 4:** Trunk rotation during the activation phase. Mean ± standard deviation values are reported for left and right rotation. A paired t-test was used to compare directions within subjects. Effect size is reported as Hedges’ g for paired designs. Confidence intervals refer to the mean difference (left minus right). Activation produced a large and statistically significant shift toward left rotation.

Rotation Parameter	Left Rotation (°)	Right Rotation (°)	t-value	p-value	Hedges’ g	95% CI (Left – Right)
Activation	62.26 ± 12.06	48.50 ± 12.00	9.95	3.7 × 10⁻¹⁵	1.15	+11.00 to +16.52

**Table 5:** Comparison between baseline and activation trunk rotation. Values are presented as mean ± standard deviation. Paired t-tests were used to assess within-subject differences. Effect sizes are reported as Hedges’ g for paired designs. Confidence intervals represent the 95% CI for the mean change (activation minus baseline). Activation induced a significant increase in left rotation and a marked reduction in right rotation.

Rotation Direction	Baseline (°), Mean ± SD	Activation (°), Mean ± SD	Mean Change (°)	t-value	p-value	Hedges’ g	95% CI of Change
Left Rotation	57.60 ± *SD*	62.26 ± *SD*	+4.67°	–4.77	9.4 × 10⁻⁶	0.55	+2.71 to +6.62
Right Rotation	57.80 ± *SD*	48.50 ± *SD*	–9.30°	+7.92	2.2 × 10⁻¹¹	–0.92	–11.64 to –6.96

**Table 6:** Total trunk rotation range of motion (ROM), expressed as the sum of left and right rotation. Values are reported as mean ± standard deviation. A paired t-test was used to compare baseline and activation ROM. Effect size is presented as Hedges’ g for paired samples. Confidence intervals refer to the 95% CI of the mean change (activation minus baseline). Activation produced a small but statistically significant reduction in total ROM.

Parameter	Baseline ROM (°) Mean ± SD	Activation ROM (°) Mean ± SD	Mean Change (°)	t-value	p-value	Hedges’ g	95% CI of Change
Total ROM (Left + Right)	115.40 ± 22.34	110.76 ± 20.96	–4.63°	2.79	0.0068	–0.32	–7.95 to –1.32

**Figure 2:**
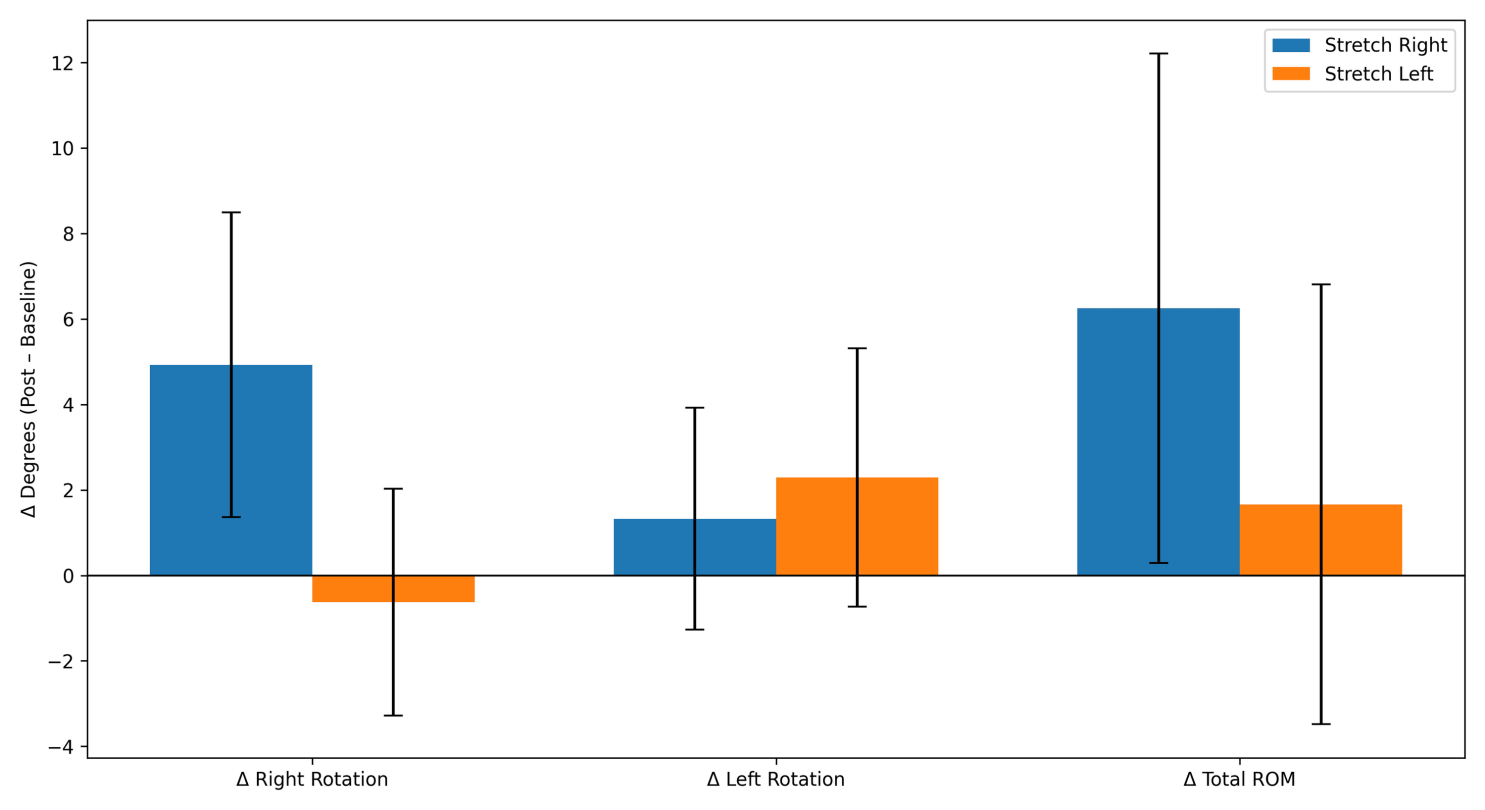
Side-specific effects of stretching on trunk rotation (Δ post–baseline). Bar chart showing changes from baseline (Δ post–baseline) in right rotation, left rotation, and total ROM after stretching. Participants are grouped by stretched side (right vs. left). Bars represent mean ± SEM. No significant differences between-group were observed.

Following stretching, trunk rotational mobility showed a partial rebalancing of the activation-induced asymmetry (Table 7, Figure 2). When changes from baseline were compared between participants stretched on the right versus the left lower limb, no significant between-group differences were observed for left rotation, right rotation, or total ROM. However, a significant difference emerged for the rotational shift (left/right), indicating a direction-specific effect of stretching on rotational balance.

**Table 7:** Effect of stretching side on trunk rotation and rotational balance. Values represent changes from baseline to post-stretch (Δ Post–Baseline). Group comparisons were performed using Welch’s t-test. Effect size is reported as Hedges’ g. Rotational shift was defined as the difference between left and right rotation (left/right); negative values indicate a shift toward the right, and positive values toward the left.

Parameter (Δ Post–Baseline)	Stretch Right (n = 39) Mean ± SD	Stretch Left (n = 34) Mean ± SD	t-value	p-value	Hedges’ g	95% CI
Δ Right rotation	+4.93 ± 10.84	+2.29 ± 9.67	0.56	0.575	0.13	−6.70 to +11.99
Δ Left rotation	+1.33 ± 9.95	+4.82 ± 10.21	−1.54	0.128	−0.36	−9.43 to +1.35
Δ Total ROM (Left / Right)	+6.26 ± 15.41	+7.11 ± 14.88	−0.24	0.809	−0.06	−8.34 to +6.64
Δ Rotational Shift Left / Right​​​​​​​)	−3.60 ± 9.12	+2.92 ± 8.47	−2.14	0.036	−0.72	−12.59 to −0.44

## Discussion

The findings of the present study contribute to the growing body of research on myofascial force transmission, which has demonstrated that forces generated within a given body region can influence the mechanical behavior of non-contiguous segments through fascial and epimuscular continuities [1,33]. Both cadaveric and in vivo studies have documented load transmission between the latissimus dorsi, the thoracolumbar fascia, and the lower limb [15,22-24]. The present work extends these observations by providing in vivo evidence obtained during a functional task, namely, trunk rotation.

Specifically, this study provides in vivo evidence supporting the existence of a functional, beyond purely anatomical, relationship between adjacent segments belonging to the posterior spiral chain (PSC). The results demonstrate that modulation of myofascial tension at the caudal level is capable of systematically altering the pattern of trunk rotation in the transverse plane.

Effect of activation: directional shift without an increase in total ROM

The most robust finding of the present study concerns the isometric activation phase of the PSC. Under this condition, a significant reduction in rightward rotation was observed, accompanied by a concomitant increase in leftward rotation, without an expansion of the overall rotational cone. This pattern suggests that muscle activation does not generically increase joint mobility but rather induces a directional redistribution of the available rotational range of motion.

From a biomechanical perspective, this behavior is consistent with the spiral myofascial chain model described by Colonna [21], according to which, during rotational movements, one chain acts as the functional agonist while the contralateral chain must effectively lengthen to permit motion. Increased active tension at one extremity of the PSC, therefore, appears to facilitate contralateral rotation while constraining ipsilateral rotation, resulting in a shift of the rotational cone rather than its expansion. Consistent with this interpretation, total trunk rotational ROM was found to be slightly, but significantly, reduced during the activation phase.

The response observed during the stretching phase further supports this conceptual framework and aligns with the work of Stecco [19], who emphasized the role of fascia as a structure capable of modulating intermuscular coordination and force distribution, rather than acting as a purely passive element. The present findings are also consistent with experimental evidence reported by Carvalhais et al. [23], who demonstrated force transmission between the latissimus dorsi and the contralateral gluteus maximus, as well as with the reviews by Wilke et al. [1], which highlighted the capacity of myofascial chains to influence global kinematics even in the absence of direct muscular continuity.

By extending these observations to the axial skeleton, the present study shows that such myofascial effects are detectable at the level of the spine during a functional task such as trunk rotation.

Effect of stretching: direction-specific response and individual variability

The stretching phase produced more complex and less uniform results compared with the activation phase. Stretching of the left lower limb was associated with a significant increase in leftward trunk rotation, whereas stretching of the right lower limb did not elicit comparable statistically significant changes in contralateral rotation. However, when the rotational balance shift (left/right) was analyzed, a relevant finding emerged: stretching induced a displacement of the rotational cone toward the treated side, independently of changes in total rotational ROM.

These findings suggest that the primary effect of stretching is not an increase in absolute joint excursion but rather a modification of the tensile balance between the two hemichains, consistent with a mechanism of selective reduction in passive tension along the PSC. The substantial inter-individual variability observed is in agreement with previous literature on fascial tissue responses to mechanical stimuli, which are known to depend on factors such as tissue stiffness, collagen organization, and individual motor strategies, as described by Schleip et al. [34].

An additional relevant aspect is that the right lower limb was consistently involved during the activation phase preceding stretching. It is therefore plausible that a persistence of activation-related effects attenuated the response to stretching on the same side, suggesting a potential interaction between active and passive tensioning mechanisms that warrants further experimental investigation.

Lateral dominance and subgroup analyses

Subgroup analyses did not reveal significant differences in baseline rotational values between males and females, indicating a largely sex-homogeneous sample. With respect to limb dominance, left-dominant participants exhibited higher mean rotational values; however, the extremely small number of left-dominant individuals substantially limited the statistical power of this comparison. In this context, the use of Hedges’ g allowed estimation of effect magnitude while reducing bias associated with small and unbalanced sample sizes, in accordance with methodological recommendations for exploratory studies.

Clinical implications

The structural complexity and three-dimensional continuity of the fascial system render this tissue particularly vulnerable to repetitive mechanical overload and maladaptive responses, which may contribute to the development of chronic musculoskeletal disorders and persistent pain syndromes. In recent years, fascia has no longer been regarded as a purely passive support structure but rather as a mechanosensitive and reactive tissue, characterized by rich innervation and the presence of contractile cellular elements, particularly myofibroblasts, capable of modifying tissue mechanical properties in response to mechanical stimuli [35].

These characteristics help explain the involvement of the myofascial system in a wide range of clinical disorders, including chronic neck pain, low back pain, adhesive capsulitis of the shoulder, and nerve entrapment syndromes. In such disorders, pain is often present not only in association with stiffness or hyperlaxity but also at end ranges of motion, suggesting an active role of fascia in modulating nociceptive perception and movement control [36-38].

The results of the present study fit within this framework, demonstrating that remote modulation of myofascial tension can produce measurable changes in trunk kinematics without the need for direct intervention at the spinal segment. In particular, the observation that both activation and stretching primarily induced a directional shift of the rotational cone, rather than an increase in total ROM, suggests that clinical interventions targeting the myofascial system may be more effective in rebalancing altered movement patterns than in pursuing a nonspecific increase in joint mobility.

From an applied perspective, these findings support a global approach to assessment and treatment, in which targeted activation or stretching strategies along specific myofascial chains may be used to influence the functional behavior of the spine and hip. This approach appears particularly relevant for injury prevention and rehabilitation, as well as within sports medicine, where the myofascial system is increasingly recognized as a key contributor to performance and mechanical load management [34].

In light of recent advances, both clinical and experimental research are increasingly focused on the dynamic adaptation and the structural and biochemical changes of fascia in response to mechanical loading. Progress in this field will require a closer integration of biomechanics, therapeutic exercise, and functional assessment tools, with the aim of developing more targeted, effective, and individualized intervention strategies.

Clinically, the present findings suggest that interventions applied to caudal segments of the PSC, through either activation or stretching, can induce measurable changes in trunk kinematics even in the absence of direct treatment of the spine. This supports a global approach to the evaluation and management of trunk rotational dysfunctions, consistent with biomechanical models attributing a central role to the thoracolumbar fascia in load transfer between the lower limbs and the spine, as described in the work of Vleeming et al. [22] and Barker [24].

The fact that both activation and stretching predominantly resulted in a redistribution of the rotational cone, rather than an increase in total ROM, further suggests that myofascial treatments may be particularly useful for restoring directional balance in movement patterns, rather than simply increasing joint mobility. This perspective may have important implications for the management of low back pain, hip dysfunctions, and disorders characterized by persistent rotational asymmetries.

Strengths and limitations

Maintaining a final sample size of 73 participants enhanced the overall robustness of the analyses and allowed more reliable exploration of several subgroup comparisons, including sex-related differences (males vs. females), lower limb dominance (right vs. left), and, in a subsequent phase, the side of stretching (right vs. left). A larger sample also helped reduce the risk of type II error, improving the stability of variability estimates and the precision of confidence intervals. Overall, the final sample size strengthens the methodological validity of the findings.

The main limitations of this study include the absence of a control group, inter-individual variability in the intensity of isometric activation, the operator-dependent nature of the stretching protocol, and the unbalanced distribution of left-dominant participants. Furthermore, it was not possible to definitively identify the specific anatomical pathway of force transmission, although the thoracolumbar fascia represents the most plausible biomechanical candidate, as suggested by anatomical and histological evidence [22].

Future studies should combine controlled experimental designs with direct measurements of fascial stiffness and neuromuscular activation to further clarify the direction-specific role of the posterior spiral chain and its clinical relevance.

## Conclusions

This study provides in vivo evidence supporting the functional relevance of the posterior spiral chain as a myofascial pathway capable of modulating trunk rotational behavior. Modulation of myofascial tension at the caudal level, through either isometric activation or targeted stretching, did not primarily alter the overall amplitude of trunk rotation but rather induced a direction-specific redistribution of the available range of motion. These findings suggest that myofascial interventions act mainly by shifting the rotational cone, influencing movement balance rather than increasing total mobility.

The observed effects during activation and stretching are consistent with current biomechanical models of spiral myofascial connectivity and support the concept that remote segments can influence axial kinematics through fascial continuity. From a clinical perspective, this highlights the potential value of addressing myofascial chains when assessing and managing trunk rotational dysfunctions, particularly in disorders characterized by asymmetrical movement patterns. Future research employing controlled designs and direct measurements of fascial mechanical properties is warranted to further clarify the mechanisms underlying direction-specific myofascial force transmission.

## Figures and Tables

**Figure 3:**
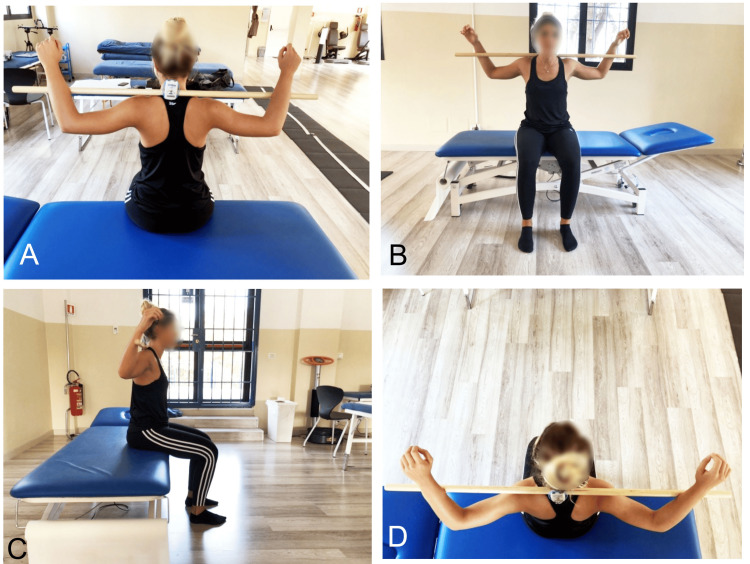
Participant testing position with the bar system and attached goniometer. A) Posterior view; B) anterior view; C) lateral view; D) superior view. Image credit: Saverio Colonna.

**Figure 4:**
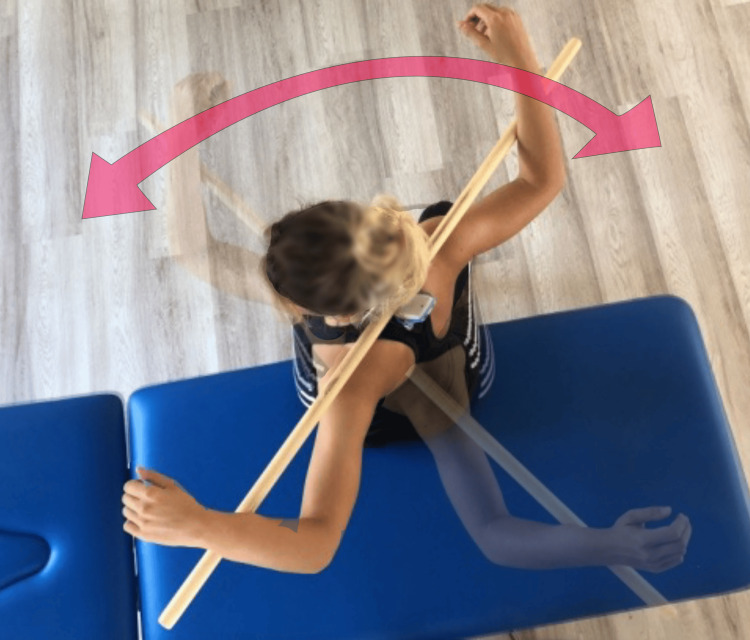
Warm-up phase execution. Trunk rotation in the transverse plane to the right and left sides (superior view). Image credit: Saverio Colonna

**Figure 5:**
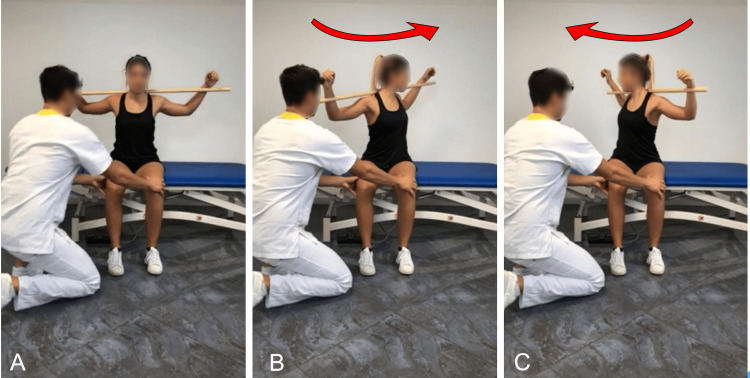
Baseline data acquisition setup. A) Examiner stabilization of both knees to minimize pelvic compensation; B) trunk rotation to the left; C) trunk rotation to the right. Image credit: Saverio Colonna

**Figure 6:**
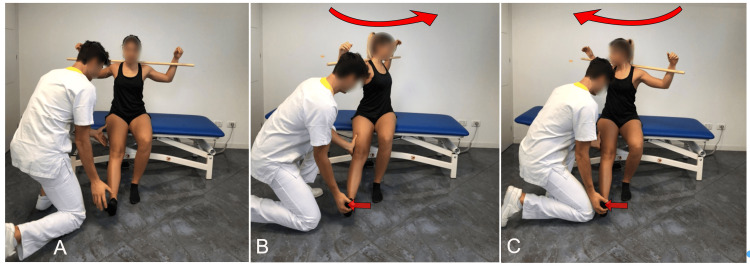
Participant and examiner positioning during the activation condition. A) Examiner hand placement to provide resistance; B) isometric contraction of the right hip abductors and external rotators with concomitant left trunk rotation, against examiner-applied counterforce on the right lower limb; (C) isometric contraction of the right hip abductors and external rotators with concomitant right trunk rotation, against examiner-applied counterforce on the right lower limb. Image credit: Saverio Colonna

**Figure 7:**
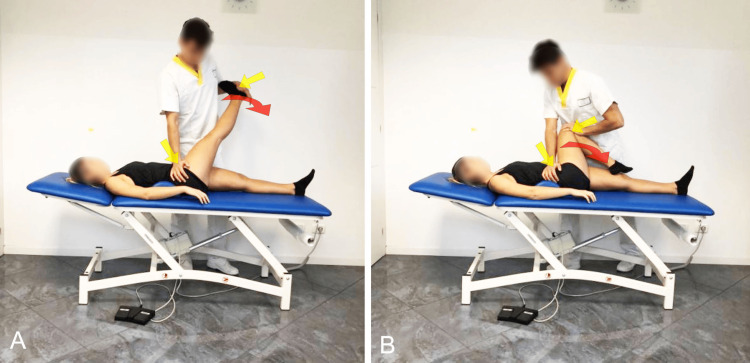
Stretching techniques for the posterior spiral chain. A) Hip flexed, adducted, and internally rotated with the knee extended; B) hip flexed, adducted, and internally rotated with the knee flexed. The red arrow indicates the force generated by the participant, whereas the yellow arrows indicate the counterforce applied by the examiner. Image credit: Saverio Colonna

## Appendices

Supplementary appendix 1

Detailed Description of the Study Procedures

Experimental protocol

The experimental protocol consisted of the following phases:

1. Warm-up: ten full trunk rotations.

2. Baseline 1 and Baseline 2: three rotations per side; the mean value was used as the reference.

3. PSC activation: isometric contraction in right hip abduction and external rotation during three rotations per side; the mean value was used for analysis.

4. Stretching was performed using a modified Muscle Energy Technique (MET), based on a sequence of submaximal isometric contractions followed by passive stretching of the targeted myofascial chain, in accordance with previously described procedures [39-41].

Specifically, METs targeting the caudal segment of the PSC on either the right or left side (randomly assigned) were performed in the supine position with lumbar support, as described in the literature [21].

5. Post-stretch assessment: trunk rotation was reassessed using the same procedure as the baseline measurements within 60 seconds after stretching.

Analyzed variables: left trunk rotation angle, right trunk rotation angle, left-right difference, total trunk range of motion (ROM).

Phase 1: Warm-up

Positioning: Participants were seated on the edge of the examination table with both feet fully supported on the floor and knees flexed at 90° (Figure 3). A wooden stick was positioned across the upper trapezius muscles behind the neck, with the distal third of the forearms in contact with the posterior aspect of the stick. The shoulders were positioned in approximately 90° of abduction and external rotation.

Execution: Participants were asked to perform ten complete trunk rotations to both right and left while holding the stick (Figure 4).

This phase aimed to prepare participants for the specific movement required during testing, pre-activate the musculature involved in trunk rotation, and identify any unusual symptoms (e.g., pain at end range). This procedure allowed participants to familiarize themselves with the task and provided adequate warming of the pelvic girdle and trunk musculature, thereby reducing potential underestimation of joint range during the initial repetitions compared with later, fully warmed movements.

Phase 2: Baseline 1

Positioning: Participants were seated on the edge of the examination table with both feet fully supported on the floor and knees flexed at 90°. A wooden stick was positioned across the upper trapezius muscles behind the neck, with the distal third of the forearms in contact with the posterior aspect of the stick. The shoulders were positioned in approximately 90° of abduction and external rotation.

The F4A inertial sensor was fixed to the midpoint of the stick and positioned approximately between the C7 and T1 vertebrae (Figure 5).

Instructions: Participants were instructed to reach their maximal trunk rotation in the transverse plane while avoiding compensatory movements.

Execution: Three rotations per side were performed in an alternating sequence, starting from a neutral position (0° rotation) (Figure 5A). By convention, the first rotation was performed toward the left (Figure 5B). The entire movement arc was required to be smooth and continuous, without jerky motions or pauses, and performed at a constant angular velocity, while minimizing compensatory strategies. The test concluded with a return to the initial neutral position. Throughout the test, an operator stabilized the lower limbs at knee level and the pelvis to minimize pelvic compensation during trunk rotation.

Phase 3: Baseline 2

Baseline 2 was performed identically to Baseline 1.

The baseline phase was repeated twice in order to obtain a less variable reference condition, using the mean of the two baseline measurements for analysis. This procedure also allowed assessment of test repeatability by verifying whether relevant differences existed between Baseline 1 and Baseline 2, thereby ensuring that any changes observed during the activation and post-stretching phases were attributable solely to the experimental interventions.

Phase 4: Activation

Positioning: Participants were seated on the edge of the examination table with their left foot supported on the floor and the left knee flexed at 90°, while the right knee was extended. The right hip was flexed and slightly externally rotated, with the right heel resting on the floor and the ankle maintained in a neutral position (Figure S4A). Upper limb and stick positioning were identical to those used during the baseline phases.

Execution: Participants were asked to perform an isometric contraction of the hip abductors and external rotators. Manual resistance was applied by the operator at the level of the knee and ankle and maintained throughout the test (Figure 6).

While maintaining the isometric contraction, participants performed trunk rotations in the transverse plane using the same execution criteria as during Baseline 1 and Baseline 2 assessments (left rotation: Figure 6; right rotation: Figure 6), with the only difference being the sustained isometric contraction in hip abduction and external rotation.

A second operator recorded trunk rotation data using the F4A system while the participant maintained the isometric contraction of the PSC. By convention, the activated chain corresponded to the left posterior spiral chain, defined as the chain linking the left shoulder to the right hip.

Phase 5: Stretching

Following a 5-minute rest period after the activation phase, MET was applied to the caudal portion of the posterior spiral chain on either the right or left lower limb according to random allocation. Stretching was then applied using two procedures based on muscle energy techniques (METs) [39-41], specifically targeting the posterior spiral chain (PSC) as proposed by Colonna and Azzena [21].

Stretching techniques (MET):

Positioning: Participants were positioned supine, with a semi-cylindrical lumbar support placed under the lumbar spine to maintain a neutral lordotic curvature.

Execution: Once the limb was positioned at the point of maximal tissue tension, the examiner instructed the participant to perform a 3-5 s submaximal isometric contraction in hip abduction against manual resistance.

This was followed by a 3-5 s relaxation phase, after which the examiner progressively increased the passive stretch by 3-5 s, advancing to the new resistance barrier. This contraction-relaxation cycle was repeated three times.

In Configuration 1, the examiner stabilized the ipsilateral iliac crest with the cranial hand, while the caudal hand controlled resistance and stretching at the ankle (Figure 7A).

In Configuration 2, the cranial hand stabilized the iliac crest, while the caudal hand applied resistance and stretch at the knee (Figure 7B).

The side of stretching was assigned using an alternating allocation procedure, starting with the left side for the first participant and subsequently alternating between right and left for each consecutive participant. This approach was adopted to ensure a balanced distribution of stretching laterality across the sample.

Phase 6: Post-Stretch Data Collection

This phase was performed identically to the baseline assessment and allowed the collection of trunk rotation range of motion data following the stretching intervention.

Phase 7: Data Processing

All data were transcribed into a spreadsheet and processed by an independent examiner who was not involved in data acquisition and was blinded to the experimental conditions.
